# Ocular findings in children with attention deficit hyperactivity disorder: A Case–Control study

**DOI:** 10.1016/j.amsu.2020.08.005

**Published:** 2020-08-15

**Authors:** Laila T. Ababneh, Mahmoud Bashtawi, Bayan F. Ababneh, Ikhlas H. Mahmoud, Mohammad Rashdan, Mohammad Zahran

**Affiliations:** a Division of Ophthalmology, Department of Special Surgery, Faculty of Medicine, Jordan University of Science and Technology, Irbid, Jordan; b Division of Psychiatry, Department of Neuroscience, Faculty of Medicine, Jordan University of Science and Technology, Irbid, Jordan; c Division of Clinical Pharmacy, Faculty of Pharmacy, Jordan University of Science and Technology, Irbid, Jordan; d King Abdullah University Hospital, Irbid, Jordan; e Faculty of Medicine, Jordan University of Science and Technology, Irbid, Jordan

**Keywords:** ADHD, Visual acuity, Ocular, Neurodevelopmental

## Abstract

**Background:**

To evaluate the differences of ocular abnormalities between children with attention deficit hyperactivity disorder and non-attention deficit hyperactivity disorder children using siblings of cases in Jordan.

**Methods:**

A case–control study of 55 children with attention deficit hyperactivity disorder, and 55 children without the disorder as a control group using siblings of cases. Examination included visual acuity, motility, anterior and posterior segments, convergence, optical coherence tomography and corneal topography.

**Results:**

Thirty-eight patients from the attention deficit hyperactivity disorder group had visual acuity better than 0.8 in both eyes; 36.4% had normal cyclorefraction, while 54.5% had mild hyperopia. Most of them did not need glasses. Tomography showed normal values with no statistically significant differences between the two groups. The near point of convergence showed significantly abnormal values in 41.9% of children with attention deficit hyperactivity disorder. Pentacam measurements showed normal values with no statistically significant differences between the two groups.

**Conclusions:**

Children with attention deficit hyperactivity disorder show significant low near point convergence compared with the study control group.

## Highlights

• ADHD is one of the most common neurodevelopmental disorders of childhood.
• It is important to rule out underlying ocular conditions.
• Children with ADHD show significant low near point convergence.
• All children with ADHD should undergo an ophthalmological assessment.

## 1 Introduction

Attention deficit hyperactivity disorder (ADHD) is one of the most common neurodevelopmental disorders of childhood, with a worldwide prevalence of 2.2%–17.8% [1]. The variability in prevalence might be due to study methodology, diagnostic criteria, population type, sample size, and cultural perceptions [[1], [2], [3], [4]]. The reported rates might vary depending on the source of the information [2,5,6].

Two studies were performed in Jordan to determine the prevalence of ADHD with differing results. The first was conducted in the north of the country and found that the prevalence was high compared to other countries with prevalence rates of combined type ADHD of 20.21%, hyperactivity–impulsivity of 9.58% and inattention of 10.83% [7]. The second study was conducted in the south of the country and found that the prevalence of ADHD was similar to that observed worldwide [8].

The emotional and behavioral problems associated with ADHD may interfere with nearly every aspect of a child's life, including family and sibling relationships, peer relationships, academic performance, planning, and task completion [2,9,10].

While it is definitely common practice to rule out any underlying neurological condition that might cause behavioral aberrations in children, it is still uncommon to refer these children to an ophthalmologist to rule out underlying ocular conditions that might interfere with the child's attention [11].

Various ocular conditions can be identified by simple screening. One of those that might have an influence on the child's behavior or his attention span during various activities like studying and reading is convergence insufficiency (CI), which was found to be significantly higher in our study group. CI can have a high impact on the patient during near viewing causing diplopia, blurred vision, asthenopia (eye strain) and slow reading [12]. This can lead to decreased school performance as was illustrated by a study by Rouse et al. [12].

This study aims to identify the ocular disease in children with ADHD, and to identify whether any specific ocular abnormality would be linked to their lack of attention. Also, this study evaluates the differences of ocular problems between children with attention deficit hyperactivity disorder and non-attention deficit hyperactivity disorder children using siblings of ADHD children in Jordan.

## 2 Material and methods

Fifty-five children diagnosed with ADHD from pediatric psychiatry clinic at KAUH were enrolled in this study as a case group, and 55 siblings of case group as a control group. All participants were assessed for all tests related to the study. ADHD was assessed using DSM-5 diagnostic criteria for cases. All ADHD cases who met the DSM-5 diagnostic criteria and checked by pediatric psychiatry consultant were eligible to participate in the study. Siblings of cases were eligible to put as control group and were confirmed not to be ADHD using the same DSM-5 diagnostic criteria and checked by same pediatric psychiatry consultant. Any participant in both groups with any comorbid psychiatric disorders, missing data and incomplete examinations were excluded.

The parents were asked to enroll their children in this study and a written consent forms were obtained from the parents. The study was approved by the Institutional Review Board Committee at our institution before data collection. It was conducted over a period of 18 months, from October 2017 until April 2019. As summarized by the Centers for Disease Control, the diagnosis of attention disorders requires six criteria to be present in a child under the age of 17 years [5,[13], [14], [15], [16], [17]]. Those include failure to give close attention to detail, trouble paying attention, not listening when spoken to, not following instructions, failing to finish schoolwork, having trouble organizing tasks and activities, avoiding tasks that require mental effort over a long period of time, and losing items necessary for various activities.

Methylphenidate (Ritalin) was prescribed according to weight and response for ADHD group. Arrangements were made to attend the pediatric ophthalmology clinic for cases and their siblings.

Visual acuity was measured in all participants in the study group. Different methods were used according to the patient's age and cooperation. In 46 patients in the ADHD group, visual acuity was obtained using a Snellen chart (a Nidek model CP-770 chart projector was used; Nidek, Tokyo, Japan), E letters and pictures, while in nine patients, vision was measured by the central, steady maintained (CSM) method because they were uncooperative and lost attention during the exam.

The anterior segments of both eyes were examined using a slit lamp (Topcon IS-80; Topcon, Tokyo, Japan). Ocular alignment and extraocular motility examinations were performed in all children. Hirschberg's test, cover-uncover, and alternate cover tests were performed. The near point of accommodation (NPA), and near point of convergence (NPC) were measured using an RAF binocular gauge rule (Clement Clarke Inc, Harlow, Essex, UK).

Cycloplegic refraction was performed in all children, 30–45 min after the last instillation of 1% cyclopentolate eye drops (total of three instillations per eye) at 10-min intervals. A Welch Allyn Retinoscope (Welch Allyn, Mississauga, Canada) was used by trained senior ophthalmology residents or by the pediatric ophthalmologist. Detailed fundus examinations were performed. Corneal topography using an Oculus Pentacam Type 70,700 (Oculus, Wetzlar, Germany) and optical coherence tomography of the macula (Nidek RS330 Retina Scan Duo; Nidek, Tokyo, Japan) were conducted.

Glasses were prescribed when appropriate, giving either the full correction to patients found to have strabismus, or according to the post-mydriatic test for subjective refraction. Patients who had ocular findings, who required glasses, or who needed another intervention including strabismus correction surgery were followed up.

Data were analyzed using SPSS software, version 20 (IBM Corp, Armonk, NY, USA). Data were presented as frequency distributions for categorical variables and mean ± standard error of the mean for continuous variables. Data was tested at a significance level of 0.05%. Pearson χ2 test was used to investigate the significance of association between categorical variables, while student's *t*-test and ANOVA were applied to examine the significance level for continuous normally distributed variables.

The work has been reported in line with the STROCSS criteria [18]. The work was submitted to research registry with the unique identifying number: researchregistry5753.

## 3 Results

A total of 110 children were included, 55 children in the ADHD group, and 55 in the control group. Table 1 shows the distribution of children by sociodemographic status, prematurity, low birth weight and delivery method.

**Table 1:** Distribution of Subjects by Sociodemographic, premature, low birth weight, delivery method and by disease status (N = 110).

	Disease status			
Variable	Controls (non-ADHD) (N = 55)		Cases (ADHD) (N = 55)	
			
Age in years, mean (SD) Range in years P-Value*	8.15 (3.47) 4–16 0.566		7.96 (3.25) 4–16	
Gender, n (%)				
Male	29	(52.7%)	43	(78.2%)
Female Total P-Value#	26 55	(47.3%) (100%) **0.000**	12 55	(21.8%) (100%)
Prematurity, n (%)				
No	55	(100%)	53	(96.4%)
Yes Total P-Value#	0 55	(0%) (100%) 0.495	2 55	(3.6%) (100%)
Low birth weight<2.5 kg, n (%)				
No	55	(100%)	46	(83.6%)
Yes Total P-Value#	0 55	(0%) (100%) **0.003**	9 55	(16.4%) (100%)
Delivery method, n (%)				
Normal	36	(65.5%)	38	(69.1%)
CS Total P-Value#	19 55	(34.5%) (100%) 0.839	17 55	(30.9%) (100%)

N: Total sample, n: Frequency, %: Percent, *: *t*-test, #: χ2.

Also, Table 2 shows the ocular performance and refraction in each group with no statistically significant difference.

**Table 2:** Ocular performances and refraction of both groups.

Control group ADHD group P-Value			
VA OD^a^ VA OD (logMAR)^a^	0.91 (0.0–1.0) 0.075 (0.0–1.0)	0.87 (0.0–1.0) 0.124 (0.0–1.0)	0.545^c^ 0.132^c^
VA OS^a^ VA OS (logMAR)^a^	0.96 (0.0–1.0) 0.53 (0.0–0.4)	0.87 (0.0–1.0) .100 (0.0–0.5)	0.084^c^ **0.025**^c^
BCVA OD^a^ BCVA OD (logMAR)^a^	0.98 (0.0–1.0) 0.04 (0.0–1.0)	0.98 (0.0–1.0) 0.058 (0.0–0.3)	1.000^c^ 0.406^c^
BCVA OS^a^ BCVA OS (logMAR)^a^	0.98 (0.0–1.0) 0.011 (0.0–0.3)	0.98 (0.0–1.0) 0.042 (0.0–0.3)	1.000^c^ **0.010**^c^
Cyclo OD^a^	+0.45 (−5.0 to + 4.5)	+0.50 (−17.0 to + 5.5)	0.905^c^
CYCLOOD, n (%)^b^ Normal Hyperopia Myopia Total	19 (34.5%) 26 (47.3%) 10 (18.2%) 55 (100%)	20 (36.4%) 30 (54.5%) 5 (9.1%) 55 (100%)	0.392^d^
Cyclo OS a	+0.46 (−4.8 to + 5.3)	+0.80 (−2.0 to + 6.3)	0.227^c^
CYCLOOs, n (%)^b^ Normal Hyperopia Myopia Total	14 (25.5%) 28 (50.9%) 13 (23.6%) 55 (100%)	19 (34.5%) 29 (52.7%) 7 (12.7%) 55 (100%)	0.127^d^

Bold p-values denote the difference was significant.

a Mean value (minimum – maximum).

b Frequency percent); VA, visual Acuity; BCVA,????? OD, right eye; OS, left eye; n, frequency.

c *t*-test.

d χ2.

Table 3 shows the values for optical coherence tomography (OCT) of the macula with no statistically significant differences between the two groups. Table 4 shows the Pentacam results for corneal topography as curvature power (KM), maximum curvature power at the front of the cornea (Kmax), and corneal astigmatism in each group with no statistically significant difference.

**Table 3:** OCT macula values for both groups.

Control group ADHD group P-Value			
OCT OD^a^	255.6 (17–402)	262.2 (101–433)	0.405^c^
OCTCFTOD, n (%)^b^ Normal Abnormal Total	39 (70.9%) 16 (29.1%) 55 (100%)	38 (69.1%) 17 (30.9%) 55 (100%)	1.000^d^
OCT OS^a^	2^d^6.6 (12^a^–550)	252.7 (175–409)	0.652^c^
OCTCFTOS, n (%)^b^ Normal Abnormal Total	41 (74.5%) 14 (25.5%) 55 (100%)	45 (81.8%) 10 (18.2%) 55 (100%)	0.489^d^

Bold p-value denote the difference was significant.

a Mean value (minimum – maximum).

b Frequency percent); OCT?? OD, right eye; OS, left eye.

c *t*-test.

d χ2.

**Table 4:** Pentacam results;KM, Kmax and Astigmatism in both groups.

Control group^a^ ADHD group^a^ P-Value^b^			
KM numerator KM denominator	43.35 (40–46) 34.09 (40–46)	42.93 (40–46) 42.91 (40–46)	0.155 0.537
KMAX numerator KMAX denominator	44.29 (42–47) 51.67 (41–51)	44.18 (41–48) 44.04 (41–47)	0.703 0.059
Astigmatism numerator Astigmatism denominator	1.08 (0.3–3.9) 1.16 (0.3–4.8)	1.06 (0.2–3.9) 1.02 (0.3–3.3)	0.872 0.371

a Mean value^a^(minimum – maximum); KM, ????? KMAX, ???

b *t*-test.

Values for near point of convergence (NPC) are summarized in Table 5 it shows a statistically significant difference.

**Table 5:** Examination of the near point of accommodation for both groups.

Control group ADHD group P-Value			
NPC PD^a^	16.49 (4–20)	11.98 (4–20)	**0.000**^c^
NPC CM^a^	6^c^58 (5–16)	9.91 (5–16)	**0.000**^c^
			
NPC, n (%)^b^ Normal Reduced Defected Total	52 (94.5%) 2 (3.6%) 1 (1.8%) 55 (100%)	32 (58.2%) 9 (16.4%) 14 (25.5%) 55 (100%)	**0.000**^d^

Bold p-value denote the difference was significant.

a Mean value (minimum – maximum).

b Frequency percent); NPC, near point of conversion; PD, ???; CM, centimeter.

c *t*-test.

d χ2.

It was found that 16.4% of children with ADHD group have reduced NPC compared to 3.6% in the control group. In addition, 25.5% of the ADHD children had abnormal NPC results compared to only 1.8% in the control group.

## 4 Discussion

Recently, an increase numbers of children are being diagnosed with ADHD worldwide. This study aims to evaluate the differences of ocular problems between children with attention deficit hyperactivity disorder and non-attention deficit hyperactivity disorder children using siblings of ADHD children in Jordan. It was found that ADHD children show significant low near point convergence compared with the control group. Many centers consider a complete ophthalmological exam mandatory for children diagnosed with ADHD. However, this is not a worldwide regular practice.

This study showed that there was no statistically significant difference in visual acuity, motility and ocular balance, fundus exam, OCT of the macula, and in Pentacam values between the ADHD group and controls. On the other hand, the near point of convergence (NPC) was significantly different between the groups. This confirms the results obtained in a study by Granet et al. in which the charts of 266 patients with CI were reviewed retrospectively, and in whom 9.8% were diagnosed with ADHD at some point. [19] The incidence of CI in the ADHD population was shown to be 15.9%, which is threefold higher than that seen in the general population (1.8–3.3%), and very similar to the results of this study.

In a case-control study of Fabian et al, a possible relationship between ADHD and undiagnosed refractive errors, concluded that both groups were comparable with regard to visual acuity at near and far distances, cycloplegic refraction and binocular function. [20] It showed that the results of NPC measurements were significantly different between the two groups, but it was clinically insignificant because the mean NPC in both groups was less than 6 cm (4.1 cm in the control group versus 5.3 cm in the ADD/ADHD group) and only 7 of those 56 patients with ADHD (12.5%) had a value greater than 6 cm.

Martin et al. compared the results of visual fields of children with ADHD before and after treatment with stimulants and showed a significant improvement from a subnormal visual field pretreatment to results comparable to the control group after treatment. [21].

The fundus images obtained by Martin et al. showed subtle morphological changes to the optic nerve and retinal vasculature, with a small disc and rim compared to controls, which indicated early disturbance of the development of neural and vascular tissue in the CNS. [21] The present study did not draw the same conclusion as it only measured OCT of the macula, where the results were comparable, with no statistically significant difference between the groups.

This study, along with other studies, showed no statistically significant differences in the presence of refractive errors or strabismus amongst children with ADHD compared with controls. [22] The authors believe that it is clinically important to diagnose and correct any misalignment or deterioration of vision in children with ADHD. It was found that, in children who needed glasses, parents did not believe that their hyperactive children could be properly examined, or that they would tolerate glasses.

The parents of the child who underwent bilateral medial rectus recession for the correction of esotropia, reported that the child had become “quieter” and “more attentive”.

This study has some limitations, first of all we have lost some cases and their siblings to do the ocular examinations because of different schedules time of ophthalmology clinic and psychiatric clinic, which resulted in a lack of a complete dataset of the visual field tests, therefore, no firm conclusions could be drawn. The second one that this study was conducted only at single hospital in Irbid; however, it is a referral tertiary teaching hospital receiving cases from all over the country. The third one that, we faced uncooperative cases whom ocular examination was not possible to be performed. It is suggested that future research should study the effect of ADHD on the visual field on a larger group and to do it on multi hospitals that is more amenable to follow-up.

## 5 Conclusion

This study concluded that children with ADHD show significant low near point convergence compared with the control group. This may prompt a policy where all children with ADHD should undergo an ophthalmological assessment.

## Author contributions

All authors (LA, MB, BA, IM, MR, and MZ) made substantial contributions to the conception and design, acquisition of data, or analysis and interpretation of data; took part in drafting the article or revising it critically for important intellectual content; gave final approval of the version to be published; and agree to be accountable for all aspects of the work.

## Provenance and peer review

Not commissioned, externally peer reviewed.

## Ethics approval and consent to participate

Institutional approval was obtained from the Institutional Review Board at Jordan University of Science and Technology. Written informed consent was obtained from the participants. This study was conducted in accordance with the Declaration of Helsinki.

## Consent for publication

Written informed consent was obtained from the patient's parents for publication.

## Availability of data and materials

The datasets generated and analyzed during the current study are available from the corresponding author.

## Conflicts of interest

The authors declare that they have no competing interests.

## Funding

This article was funded by the Deanship of Research at 10.13039/501100004035Jordan University of Science and Technology to recruit the participants and investigate them ta (Grant number: 20170233).
